# Mismatch Negativity in Schizophrenia – A Systematic Review and Meta-Analysis

**DOI:** 10.1192/bjo.2026.11113

**Published:** 2026-06-30

**Authors:** Laura Convertino, Kuba Olszak, Ekaterina Shatalina, Katherine Beck

**Affiliations:** ^1^South London and the Maudsley NHS Foundation Trust, London, United Kingdom; ^2^University of Edinburgh, Edinburgh, United Kingdom; ^3^Psychosis Studies, Institute of Psychiatry, Psychology and Neuroscience, King’s College London, London, United Kingdom

## Abstract

**Aims:**

Schizophrenia is associated with abnormalities in mismatch negativity (MMN), an event-related potential linked to sensory memory and information-processing deficits. MMN has been widely studied as a biomarker for disease progression. This review and meta-analysis examines the magnitude of MMN abnormalities in schizophrenia and explores factors associated with these effects. In addition, this study evaluates a large language model approach for automated data extraction, with potential applications for future meta-analyses.

**Methods:**

MEDLINE, Embase and PsycINFO databases were searched from inception to 14 November 2025. A total number of 2493 articles investigating auditory MMN in patients with an established diagnosis of schizophrenia, first-episode psychosis, high-risk groups, and first-degree relatives was identified. Manual data extraction was compared to an automated data extraction using OpenAI’s GPT5 model. The mean effect size of auditory MMN in patients compared to controls was calculated. Subgroup analyses for deviant type and auditory paradigm were conducted. The effects of age, sex, illness duration, symptom severity and antipsychotic dose were analysed.

**Results:**

A total of 140 studies were included in the analysis. Compared to the controls, patients with schizophrenia showed reduced auditory MMN amplitudes (g=0.72, 95% CI [0.63–0.82]). MMN impairment increased significantly (p<0.0001) across illness stages from high-risk individuals (g=0.37) to first-episode psychosis (g=0.50) and chronic schizophrenia (g=0.85). Subgroup analyses by deviant type and auditory paradigm showed no statistically significant differences in effect sizes. Auditory MMN amplitude was not significantlyreduced among first-degree relatives. Meta-regression analyses found that both antipsychotic dose (p<0.0001) and illness duration (p<0.0001) significantly moderated MMN effect sizes, while sex, symptom scales’ scores and patient age were not significant modulators. OpenAI’s GPT5 model accurately extracted data across 95% of fields.

**Conclusion:**

This large meta-analysis confirms that auditory MMN amplitude is reduced in schizophrenia and modulated by both duration of illness and antipsychotic dose. This is present in high-risk groups, intensifying with progression to first-episode psychosis and chronic schizophrenia. These findings reinforce MMN as a potential biomarker of neurophysiological dysfunction in schizophrenia across stages of illness. Additionally, automated data extraction provided promising feasibility for accelerating future large-scale meta-analytic workflows.

